# Decreased Function of Delayed Recall in Non-demented Elderly Subjects With Apolipoprotein E ε4 Allele

**DOI:** 10.3389/fnagi.2019.00071

**Published:** 2019-03-29

**Authors:** Lijun Wang, Xiaoli Pan, Guoqiang Fei, Changpeng Wang, Wenbin Wan, Shaoming Sang, Hui Wang, Zhiliang Wang, Chunjiu Zhong

**Affiliations:** ^1^Department of Neurology, Zhongshan Hospital, State Key Laboratory of Medical Neurobiology, Institutes of Brain Science, Fudan University, Shanghai, China; ^2^Regional Health Service Center of Xujiahui, Shanghai, China

**Keywords:** APOE, MMSE, cognitive ability, non-demented elderly, delayed recall

## Abstract

Apolipoprotein E (APOE) is the major genetic risk factor for late-onset Alzheimer’s disease (AD). Inconsistent results about the role of APOE ε4 alleles on cognitive decline of community non-dementia elderly have been reported. This study aimed to examine the relationship between APOE ε4 allele and cognitive abilities in the subjects aged 60 years or above from a community in Shanghai, China. A total of 1445 participants voluntarily accepted the analysis of APOE genotype and global cognitive assay using the Mini Mental Status Evaluation (MMSE). There were no significant differences in total MMSE scores between APOE ε4 carriers and non-carriers. In addition, the performances of orientation, registration, attention, calculation, and language had no significant differences between subjects with and without APOE ε4 allele. However, stratified analysis showed that the performance of delayed recall in subjects with APOE ε4 allele was inferior to that in non-ε4 carriers (*p* = 0.041). Further, the multiple linear regression analysis showed the significant correlations between the presence of APOE ε4 allele and the scores of the delayed memory subdomain if age, gender, and education were adjusted but no significant correlations if the related factors were not adjusted. The results indicate that significant impact of APOE ε4 allele only on the delay memory but not on global or other sub-domains of cognitive abilities.

## Introduction

Apolipoprotein E (APOE) is the most recognizable genetic risk factor for late-onset Alzheimer’s disease (AD) (Corder et al., 1993; Poirier et al., 1993). The APOE gene is polymorphic, with three different alleles (ε2, ε3, and ε4), which engenders six different genotypes (ε2/ε2, ε2/ε3, ε2/ε4, ε3/ε3, ε3/ε4 and ε4/ε4). It has been reported that APOE and its receptors, such as neuronal LDL receptor-related protein 1 (LRP1), play an important role in Aβ production and clearance in the brain (Kounnas et al., 1995; Tachibana et al., 2019). Mounting evidences have also demonstrated that individuals with ε4 alleles are inclined to developing AD than those without ε4 alleles, whereas the ε2 allele is associated with protection against AD compared to the ε3 allele (Bu, 2009).

However, the effect of APOE ε4 allele on cognitive function in non-demented adults remains controversial. Many studies have found that APOE ε4 carriers had inferior cognitive performances as compared to non-ε4 carriers in non-demented individuals (Small et al., 1998; Hofer et al., 2002; Bretsky et al., 2003; Greenwood et al., 2005; Caselli et al., 2007, 2009; Risacher et al., 2013). On the contrary, some researchers reported that the presence of APOE ε4 allele had no significant impact on cognitive tests in non-demented people, especially in very old elders (Bondi et al., 1995; Negash et al., 2009). This inconsistent results drive us to design the current study in order to assess the impacts of APOE ε4 allele on cognitive abilities using Mini Mental Status Evaluation (MMSE) scoring in a non-demented population aged 60 years or older in Shanghai, China.

## Materials and Methods

### Study Design and Subjects

Participants who live in Xujiahui Street Community, Xuhui District, Shanghai, China were voluntarily recruited except subjects with illiterate from the Physical Examination Department at the Regional Health Service Center of Xujiahui from April 1, 2012 to December 31, 2014. All Participants were aged 60 years or older. Information of basic demographics, the history of diseases and medications were collected by trained investigators. The demographic information of the participants was shown in Table 1. The study was approved by the Committee on Medical Ethics of Zhongshan Hospital, Fudan University. All subjects gave written informed consent in accordance with the Declaration of Helsinki.

**Table 1:** Baseline characteristics of participants by APOE ε4 zygosity.

Characteristics	Total (*n* = 1445)	ε4 Carriers (*n* = 268)	ε4 Non-carriers (*n* = 1177)	*x*^2^ *t z*	*p-value*
Age	71.95 (5.65)	71.93 (5.62)	71.96 (5.66)	-0.059	0.953
Gender (female), %	755 (52.2)	139 (51.87)	616 (52.34)	0.019	0.889
BMI (kg/m^2^)	23.91 (3.25)	23.52 (3.27)	24.00 (3.24)	2.192	**0.029**
Education	11.42 (3.51)	11.71 (3.54)	11.35 (3.50)	-1.658	0.097
MMSE total scores	28.57 (1.55)	28.44 (1.63)	28.60 (1.54)	-1.328	0.184
Orientation	9.92 (0.35)	9.90 (0.37)	9.92 (0.35)	-0.994	0.320
Registration	2.97 (0.18)	2.96 (0.19)	2.97 (0.17)	-0.489	0.625
Attention and calculation	4.63 (0.76)	4.59 (0.79)	4.64 (0.75)	-0.961	0.336
Recall	2.48 (0.76)	2.40 (0.78)	2.49 (0.75)	-2.046	**0.041**
Language	8.57 (0.78)	8.57 (0.77)	8.57 (0.78)	-0.042	0.966
HTN, %	724 (50.1)	132 (49.25)	592 (50.30)	0.095	0.758
DM, %	249 (17.2)	44 (16.42)	205 (17.42)	0.153	0.696
FBG (mmol/L)	5.77 (1.51)	5.73 (1.65)	5.78 (1.48)	-1.298	0.194
TC (mmol/L)	5.21 (1.04)	5.25 (0.95)	5.20 (1.06)	-0.697	0.486
TG (mmol/L)	1.50 (0.94)	1.46 (0.87)	1.51 (0.96)	-0.961	0.337
UA (μmol/L)	324.40 (78.37)	318.00 (77.25)	325.86 (78.59)	-1.442	0.149
Hb (g/L)	138.18 (13.06)	138.17 (13.19)	138.19 (13.04)	-0.006	0.995
ALT (U/L)	22.67 (11.01)	21.18 (11.09)	23.01 (10.97)	-3.260	**0.001**
Cr (mmol/L)	67.36 (21.30)	66.25 (17.04)	67.61 (22.16)	-0.383	0.702


*BMI, body mass index; MMSE, mini-mental state examination; HTN, hypertension; DM, diabetes mellitus; FBG, fasting blood glucose; TC, total cholesterol; TG, triglycerides; UA, uric acid; Hb, hemoglobin; ALT, alanine aminotransferase; Cr, creatinine. Bold values denote significant difference. For gender, hypertension, diabetes mellitus, values were expressed as number (%). Data were presented as mean ± standard deviation (SD)*.

### Cognitive Ability Assay

The global cognitive abilities were measured by the MMSE test with scores ranging from 0 (severe impairment) to 30 points (no impairment). The MMSE test includes items on several cognitive domains such as orientation (10 points), registration (3 points), attention and calculation (5 points), recall (3 points), language (9 points). Non-demented subjects were defined according to their education background as the followings: individuals with 1–6 years of education background had more than 21 scores; individuals with more than 6 years of education background had more than 24 scores. Participants who failed to meet the above criteria were excluded.

### Blood Biochemical Investigations

Fasting blood glucose (FBG), total cholesterol (TC), triglycerides (TG), uric acid (UA), hemoglobin (Hb), alanine aminotransferase (ALT), and creatinine (Cr) were investigated to acquire the general health condition of individuals.

### APOE Genotyping

Blood samples were obtained and frozen at –80°C until the determination of APOE phenotypes. The purification of human genomic DNA was isolated from peripheral blood using a genome extraction kit (TIANGEN Biotech Co., Beijing, China). APOE genotypes (rs429358 and rs7412) were detected using the ABI real-time Taqman SNP genotyping assay (ABI, Life Technologies, Carlsbad, CA, United States) in accordance with the manufacturer’s instructions. Both SNP112 (rs429358) and SNP 158 (rs7412) are done separately in 2 plates for the same samples and the results were combined for genotypes. Briefly, 1 μL of the DNA samples were added to 10 μL of Taqman Master Mix, 0.25 μL of Taqman SNP genotyping assay probe (rs429358 or rs7412) and 8.75 μL of water. The PCR conditions were denaturation at 95°C for 5 min, followed by 40 cycles of 95°C for 10 s, 60°C for 45 s. Finally, the genotypes are combined for APOE genotype of individuals. Subjects with APOE ε2/ε4, ε3/ε4 and ε4/ε4 made up to APOE ε4 carriers group, and subjects with APOE ε2/ε2, ε2/ε3 and ε3/ε3 made up to APOE ε4 non-carriers group.

### Statistical Analysis

Statistical analyses were performed using SPSS software (version 21.0; IBM SPSS). All *p* values were two-sided with statistical significance level set at <0.05. Demographic data were compared between APOE ε4 carriers and non-ε4 carriers using Student’s *t*-test or nonparametric Mann–Whitney *U* test for continuous variables. Chi-square test was utilized for dichotomous variables (such as gender, comorbidities, genotype distribution). Linear regression was used to evaluate the relationship between APOE ε4 status and cognitive subdomains assayed by different items of MMSE scoring.

## Results

### Clinical Characteristics of the Subjects Stratified by APOE Alleles

A total of 1445 subjects (including 268 APOE ε4 carriers and 1177 APOE ε4 non-carriers) were enrolled in the present study. The average age was 71.95 ± 5.65 (range = 63–91) years, and 52.2% were female.

There were no significant differences in age (*z* = -0.059, *p* = 0.953), gender (*x*^2^ = 0.019, *p* = 0.889), education levels (*z* = -1.658, *p* = 0.097), and comorbidities such as hypertension (*x*^2^ = 0.095, *p* = 0.758) and diabetes mellitus (*x*^2^ = 0.153, *p* = 0.696) between APOE ε4 carrier and non-ε4 carrier groups. Also, there were no statistical differences in the levels of FBG (*z* = -1.298, *p* = 0.194), TC (*z* = -0.697, *p* = 0.486), TG (*z* = -0.961, *p* = 0.337), UA (*z* = -1.442, *p* = 0.149), Hb (*z* = -0.006, *p* = 0.995), and Cr (*z* = -0.383, *p* = 0.702) between the two groups. APOE ε4 carriers exhibited significantly lower values for BMI (*t* = 2.192, *p* = 0.029), ALT (*z* = -3.260, *p* = 0.001). Detailed demographic information on the participants was shown in Table 1.

The phenotype and allele frequencies of APOE were showed in Table 2. 18.55 percent of the study population was APOE ε4 carriers of whom 1.73% was APOE homozygous and 16.82% was APOE ε4 heterozygous. The frequencies of APOE ε2, ε3 and ε4 alleles were 9.62, 80.24, and 10.14%, respectively.

**Table 2:** Genotypic distribution and allele frequencies of APOE polymorphism in the sample (*n* = 1445).

Genotype	*n*	%
ε2-ε2	24	1.66
ε2-ε3	180	12.46
ε2-ε4	50	3.46
ε3-ε3	973	67.34
ε3-ε4	193	13.36
ε4-ε4	25	1.73
**Allele**		
ε2	278	9.62
ε3	2319	80.24
ε4	293	10.14


*The genotypic frequencies are indicated in absolute values and percentage*.

### APOE ε4 Allele and Cognitive Abilities

There were no significant differences in total MMSE scores (*z* = -1.328, *p* = 0.184) between APOE ε4 carriers and non-ε4 carriers. The scores of delayed recall (total score is 3 scores) were significantly lower in APOE ε4 carriers as compared to those in non-ε4 carriers (*z* = -2.046, *p* = 0.041). The scores of orientation (total score is 10 scores), registration (total score is 3 scores), attention and calculation (total score is 5 scores), and language (total score is 9 scores) in subjects with APOE ε4 allele were not significantly different with those in non-ε4 carriers (*z* = -0.994, *p* = 0.320 for orientation; *z* = -0.489, *p* = 0.625 for registration; *z* = -0.961, *p* = 0.336 for attention and calculation; *z* = -0.042, *p* = 0.966 for language). The multiple linear regression analysis showed the significant correlations between the presence of APOE ε4 allele and the scores of the delayed memory subdomain if education were adjusted (Model 6, *t* = -2.091, *p* = 0.037) or if age, gender, and education were adjusted (Model 7, *t* = -2.105, *p* = 0.035) or if age, gender, education, and BMI were adjusted (Model 8, *t* = -2.097, *p* = 0.036) or if age, gender, education, BMI, and biochemical factors (HTN, DM, FBG, TC, TG, UA, Hb, ALT, Cr) were adjusted (Model 9, *t* = -2.162, *p* = 0.031). However, there were no significant differences between the presence of APOE ε4 allele and the scores of the delayed memory subdomain if all factors were unadjusted (Model 1) or only age (Model 2) or only gender (Model 3) or only BMI (Model 4) or age and gender were adjusted (Model 5, Table 3).

**Table 3:** Results of analyses of association between APOE ε4 allele carriers versus non-carriers and dimensions underlying the MMSE (unadjusted and adjusted linear regression).

MMSE Subscore	Model 1	Model 2	Model 3	Model 4	Model 5	Model 6	Model 7	Model 8	Model 9
Orientation	-0.023 (-0.067 to 0.026)	-0.023 (-0.067 to 0.025)	-0.023 (-0.067 to 0.026)	-0.024 (-0.068 to 0.025)	-0.023 (-0.067 to 0.025)	-0.024 (-0.068 to 0.025)	-0.024 (-0.068 to 0.025)	-0.025 (-0.069 to 0.024)	-0.027 (-0.071 to 0.022)
	*t* = -0.882, *p* = 0.378	*t* = -0.888, *p* = 0.375	*t* = -0.881, *p* = 0.378	*t* = -0.919, *p* = 0.358	*t* = -0.889, *p* = 0.374	*t* = -0.927, *p* = 0.354	*t* = -0.919, *p* = 0.358	*t* = -0.957, *p* = 0.339	*t* = -1.029, *p* = 0.304
Registration	-0.013 (-0.029 to 0.018)	-0.013 (-0.029 to 0.018)	-0.013 (-0.029 to 0.018)	-0.015 (-0.031 to 0.017)	-0.013 (-0.029 to 0.018)	-0.016 (-0.031 to 0.016)	-0.016 (-0.031 to 0.016)	-0.018 (-0.032 to 0.015)	-0.020 (-0.032 to 0.015)
	*t* = -0.489, *p* = 0.625	*t* = -0.496, *p* = 0.620	*t* = -0.491, *p* = 0.624	*t* = -0.583, *p* = 0.560	*t* = -0.499, *p* = 0.618	*t* = -0.618, *p* = 0.537	*t* = -0.608, *p* = 0.543	*t* = -0.690, *p* = 0.490	*t* = -0.747, *p* = 0.455
Attention and calculation	-0.025 (-0.149 to 0.053)	-0.025 (-0.149 to 0.052)	-0.025 (-0.150 to 0.053)	-0.024 (-0.149 to 0.054)	-0.025 (-0.149 to 0.051)	-0.029 (-0.158 to 0.043)	-0.029 (-0.157 to 0.043)	-0.028 (-0.156 to 0.044)	-0.031 (-0.160 to 0.040)
	*t* = -0.934, *p* = 0.350	*t* = -0.947, *p* = 0.344	*t* = -0.940, *p* = 0.347	*t* = -0.920, *p* = 0.358	*t* = -0.956, *p* = 0.339	*t* = -1.127, *p* = 0.260	*t* = -1.114, *p* = 0.265	*t* = -1.092, *p* = 0.275	*t* = -1.175, *p* = 0.240
Recall	-0.049 (-0.195 to 0.005)	-0.049 (-0.195 to 0.004)	-0.049 (-0.195 to 0.005)	-0.050 (-0.197 to 0.004)	-0.049 (-0.195 to 0.005)	-**0.055 (-0.205 to 0.007)**	-**0.055 (-0.205 to-0.007)**	-**0.055 (- 0.205 to- 0.007)**	-**0.056 (-0.208 to-0.010)**
	*t* = -1.864, *p* = 0.063	*t* = -1.875, *p* = 0.061	*t* = -1.858, *p* = 0.063	*t* = -1.882, *p* = 0.060	*t* = -1.870, *p* = 0.062	***t* = -2.091, *p* = 0.037**	***t* = -2.105, *p* = 0.035**	***t* = -2.097, *p* = 0.036**	***t* = -2.162, *p* = 0.031**
Language	-0.002 (-0.108 to 0.099)	-0.003 (-0.106 to 0.096)	-0.003 (-0.108 to 0.098)	-0.004 (-0.112 to 0.095)	-0.003 (-0.107 to 0.094)	-0.016 (-0.128 to 0.066)	-0.015 (-0.125 to 0.066)	-0.016 (-0.128 to 0.064)	-0.014 (-0.124 to 0.068)
	*t* = -0.088, *p* = 0.930	*t* = -0.104, *p* = 0.917	*t* = -0.097, *p* = 0.922	*t* = -0.167, *p* = 0.868	*t* = -0.120, *p* = 0.905	*t* = -0.626, *p* = 0.532	*t* = -0.606, *p* = 0.545	*t* = -0.646, *p* = 0.518	*t* = -0.573, *p* = 0.567


*Model 1: unadjusted; Model 2: adjusted by age; Model 3: adjusted by gender; Model 4: adjusted by BMI; Model 5: adjusted by age, gender; Model 6: adjusted by education; Model 7: adjusted by age, gender, education; Model 8: adjusted by age, gender, education, BMI; Model 9: adjusted by age, gender, education, BMI, HTN, DM, FBG, TC, TG, UA, Hb, ALT, Cr. Bold values denote significant difference. Data were presented as OR 95% CI*.

## Discussion

Increasing evidence suggested that APOE isoforms differentially regulate synaptic plasticity and repair by redistributing lipids to regenerating axons and to Schwann cells during remyelination (Buttini et al., 2002; Chen et al., 2010; Zhao et al., 2018). Emerging observations suggested that APOE ε4 contributes to AD pathogenesis by initiating and accelerating Aβ accumulation, aggregation, and deposition in the brain (Ellis et al., 1996; Holtzman et al., 2012). In addition, neuroimaging studies have showed shrinkage in hippocampal volume in APOE ε4 carriers with mild cognitive impairment (MCI) and AD compared to APOE ε4 non-carriers (Plassman et al., 1997; Liu et al., 2013), which correlated with reduced cognition memory performance (Lind et al., 2006). Interestingly, researchers have found that APOE ε4 has different effects on cognition decline depending on age (Small et al., 2004; Lind et al., 2006). Together, these mounting findings suggest that APOE ε4 genotypes exist multiple effects on risk of AD.

The current results showed that APOE ε4 allele does not exhibit significant effect on global cognitive performance as measured by the MMSE scoring among the non-demented elderly. However, the further analysis of the sub-items of MMSE showed that subjects with APOE ε4 allele performed worse in delayed recall subdomain, but not in other sub-items of MMSE scoring. Furthermore, the analysis by linear regression model showed that the presence of APOE ε4 allele was significantly correlated with delayed recall after adjustment for education. These results are in agreement with previous studies that highlight the specific effect of APOE ε4 on delay memory but not on global cognitive function (Jorm et al., 2007; De Blasi et al., 2009; Quintino-Santos et al., 2015).

However, some literatures reported inconsistent findings. It has been argued that APOE ε4 allele had a significant effect on cognitive performance (Packard et al., 2007; Negash et al., 2009; Salmon et al., 2013). One of the most important methodologic concerns of studies is age difference of subjects across various studies. Another important reason is the different environmental, social, and biological backgrounds of the populations examined. Thirdly, individuals with more education have better cognitive performance than less educated population. A higher level education may protect against cognitive deficit in late life (Katzman, 1993).

The evaluation of MMSE scores depended on the subject’s age and on educational level. In the current study, we examined subjects who were randomly selected to participate in a routine physical examination, whose age was 60 years or above. Importantly, there was no significant difference in age between APOE ε4 carriers and non-ε4 carriers. Further, the mean years of education were 11.71 ± 3.54 for APOE ε4 carriers and 11.35 ± 3.50 years for non-ε4 carriers. Further exploration is needed to evaluate the relationship between the APOE genotype and cognitive performance as measured by the MMSE.

In summary, our study indicates significant effect of APOE ε4 allele on delayed memory but not on abilities of other cognitive sub-domains or global cognition in non-demented old community individuals. The decline of delayed memory associated with APOE ε4 allele may be an available phenotype of early AD and the relationship with specific pathophysiological alterations such as brain β-amyloid and Tau aggregation and glucose hypometabolism should be further clarified by prospective and longitudinal study.

## Author Contributions

CZ conceived and designed the studies. LW wrote the article. LW, XP, GF, CW, WW, SS, HW, and ZW performed the research. LW and XP analyzed the results.

## Conflict of Interest Statement

The authors declare that the research was conducted in the absence of any commercial or financial relationships that could be construed as a potential conflict of interest.
